# Concurrent Umbilical Endometriosis and Umbilical Hernia: A Rare Case

**DOI:** 10.7759/cureus.112211

**Published:** 2026-07-07

**Authors:** Baraka S Mshango, Alex J Makalla, Asafu M Munema

**Affiliations:** 1 Department of Obstetrics and Gynaecology, Sokoine Regional Referral Hospital, Lindi, TZA; 2 Department of Surgery, Sokoine Regional Referral Hospital, Lindi, TZA; 3 Department of Pathology, Ocean Road Cancer Institute, Dar es Salaam, TZA

**Keywords:** endometriosis, umbilical, umbilical endometriosis, umbilical hernia, umbilical swelling

## Abstract

Endometriosis of the umbilicus is uncommon. Presenting symptoms are nonspecific and may mimic other diseases of the umbilicus, such as Sister Mary nodule, granuloma, melanoma, and umbilical hernia. Umbilical endometriosis and umbilical hernia can rarely occur together, and their clinical features can easily be confused. We present the case of a 34-year-old woman with a history of two cesarean deliveries attended at our center who complained of a reducible umbilical swelling of gradual onset, more marked during coughing and straining. Thereafter, she noticed another small dark bluish swelling developing on the umbilicus, which increased progressively over time. Near and during menstruation, the swelling became larger and more painful, and was later accompanied by cyclic umbilical bleeding. Physical examination revealed a darkish, firm umbilical swelling, which was non-tender and measured about 3 cm × 2 cm, as well as an abdominal wall defect measuring about 3 cm in the umbilical area. She underwent excision of the umbilical mass followed by surgical repair. The excised specimen was sent for histopathology, which confirmed an endometriosis of the umbilicus. Umbilical swelling has several differential diagnoses, which can be either primary or metastatic disease of the umbilicus. Concurrent umbilical endometriosis and umbilical hernia are rare, and their clinical features can easily be confused, causing difficulties in making an accurate diagnosis. Cyclic umbilical swelling, pain, and umbilical bleeding should raise the suspicion of umbilical endometriosis. This case report emphasizes the importance of thorough history taking, physical examination, and histopathological examination for all women presenting with ambiguous umbilical swelling.

## Introduction

Endometriosis is the presence of endometrial tissue outside the uterus, which can grow and be stimulated by ovarian hormones. This growth usually induces inflammation, pain, and, occasionally, abnormal scarring. Endometriosis may affect up to 10% of women of reproductive age. Although it commonly occurs in pelvic organs such as the ovary, it can also occur in extrapelvic organs, including the large bowel, bladder, and ureter [1,2].

Endometriosis can be categorized into primary and secondary. Primary endometriosis occurs spontaneously, while secondary endometriosis occurs following surgical interventions that penetrate or open the uterine cavity, such as cesarean section, myomectomy, and hysterectomy. It has also been reported in some cases following an episiotomy and amniocentesis [2,3].

Umbilical endometriosis is a rare form of extrapelvic endometriosis, with 0.5% to 1% of extrapelvic endometriosis occurring in the umbilicus. Umbilical hernia describes a structural defect of the abdominal wall in the umbilical area. It affects about 14% of all cases of abdominal hernia, but it can reach up to 60% in surgically naïve adults [4,5].

Umbilical endometriosis and umbilical hernia very rarely occur together, and their clinical features are easily confused, which complicates accurate diagnosis. Clinicians need to pay attention to patient history and physical examination. Radiological evaluation using imaging such as ultrasonography, MRI, and CT may aid in making the correct diagnosis. However, diagnosis should be confirmed by the presence of ectopic endometrial stroma, glands, and usually hemosiderin-laden macrophages during histological examination after surgical excision or fine-needle aspiration [6].

Management options may include hormonal therapy or wide surgical excision. Surgical intervention is the gold-standard treatment option as it is curative with a low recurrence rate of less than 5%, and it allows for histopathological examination [7].

## Case presentation

A 34-year-old woman, G2P2, who delivered twice by cesarean section, presented at our center with an umbilical swelling for three years. The swelling was of gradual onset, was initially reducible but increased in size over time, and was more marked when coughing and during activities that strain the abdomen. Four months after the onset of this swelling, she noticed another small darkish swelling that developed on the umbilicus, which increased progressively over time.

She also reported that during the menstrual period, the umbilicus enlarged, darkened, and became painful. Three months before seeking care at our facility, she had experienced cyclic umbilical bleeding. She occasionally had constipation, but no vomiting or diarrhea. During the course of this illness, she was attended at different primary health facilities and was managed as a patient with an umbilical hernia with medical management. Later, she attended an alternative medicine clinic, where she was treated with herbal medication with subtle improvement. She was a nonsmoker and a nonalcoholic.

On examination, the patient was fully conscious (Glasgow Coma Scale score: 15/15), afebrile, not jaundiced, not pale, and with no lower limb edema. Vital signs were a blood pressure of 138/80 mmHg; a heart rate of 89 beats/minute, regular; a respiratory rate of 20 breaths/minute, regular; an axillary temperature of 37°C; and oxygen saturation of 99% on room air. The patient’s body weight was 78 kg, and height was 160 cm; her BMI was 30.5 kg/m² (classified as obese).

**Figure 1:**
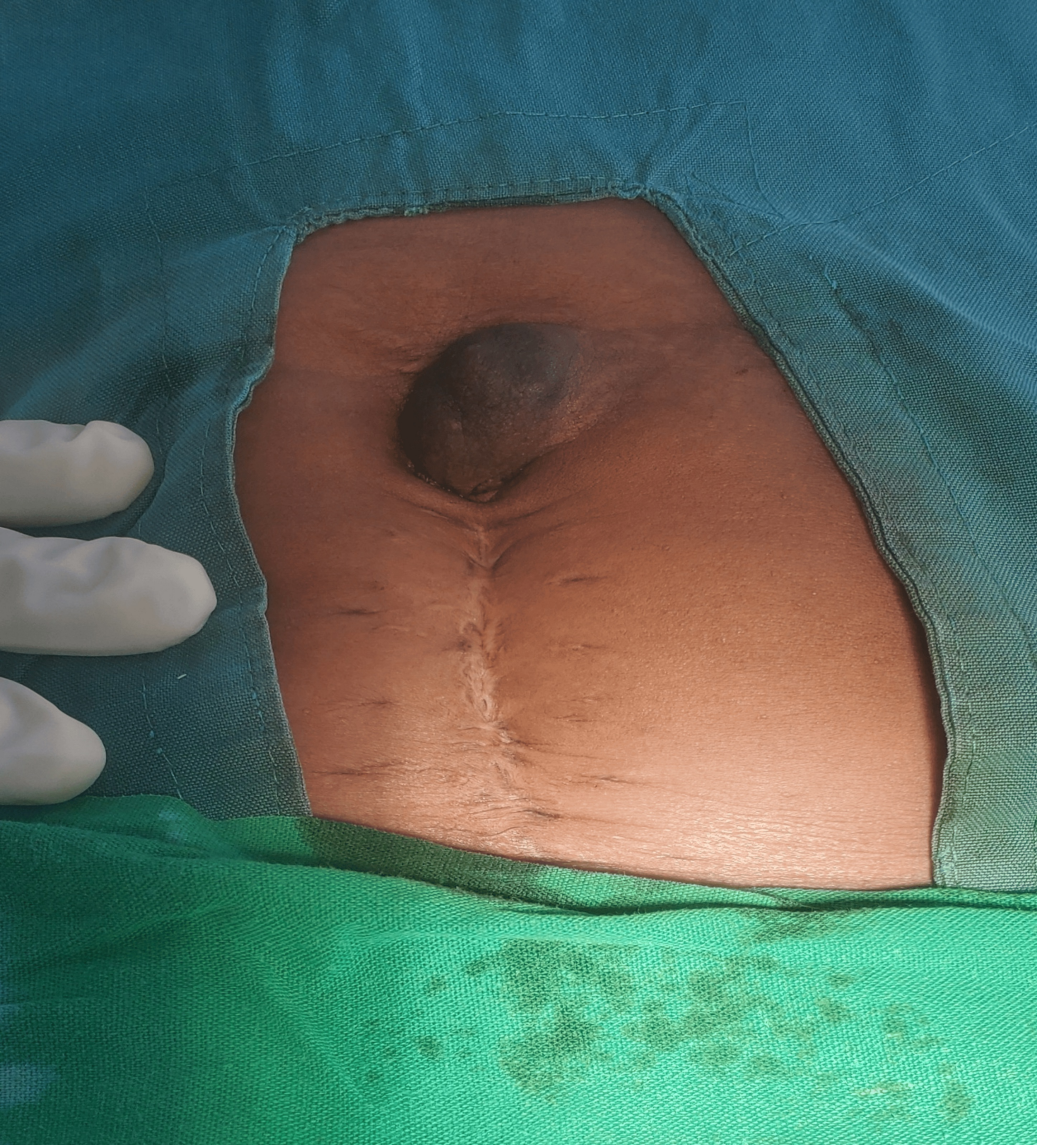
Umbilical swelling before surgical excision.

Abdominal examination revealed a mildly distended abdomen with a dark, bluish, firm umbilical swelling, non-tender, measuring about 3 cm × 2 cm, and an abdominal wall defect (hernia defect) in the umbilical area with a hernia orifice of about 3 cm. There was a subumbilical midline surgical scar. Normal bowel sounds were heard on auscultation. The liver and spleen were not palpable. The pelvic examination was normal, and there were no remarkable findings on other systems (Figure 1).

On laboratory investigations, hemoglobin was 13.1 g/dL (reference range: 12.0-15.9), and platelet count was 366 U/L (platelet count: 155-366). Abdominopelvic ultrasound revealed a small abdominal wall defect (hernia defect). There were no other lesions or abnormalities in the intra-abdominal organs or abdominal wall lesions.

**Figure 2:**
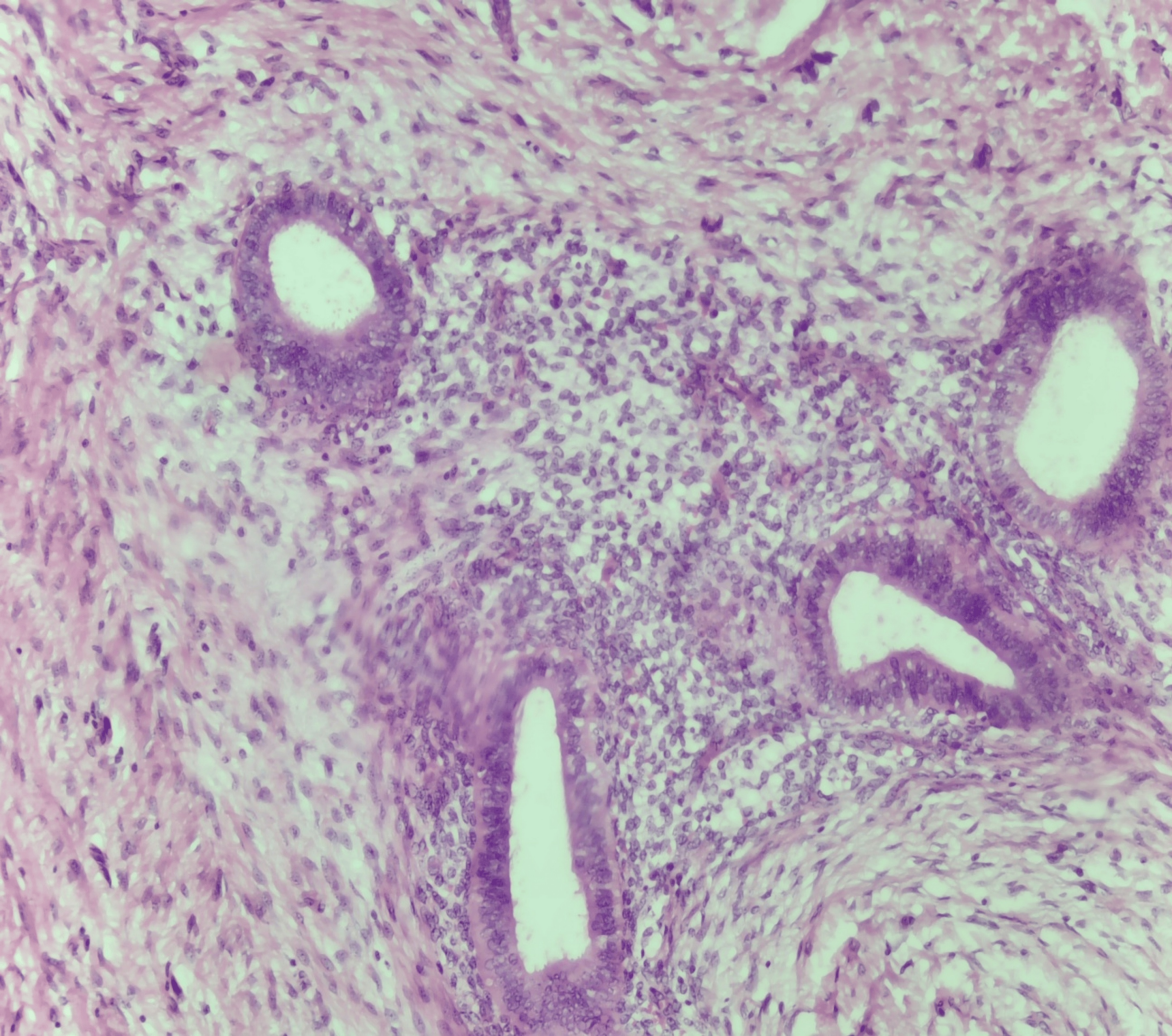
Histopathology section of the removed umbilical mass showing features of endometrial tissue (hematoxylin and eosin staining at 10× original magnification).

The diagnosis of umbilical endometriosis and hernia was reached. The patient underwent surgical excision followed by repair. The excised specimen was sent for histopathological evaluation, which confirmed the diagnosis (Figure 2). No complications were observed in the six months of follow-up after surgery.

## Discussion

We report a case of a 34-year-old woman with a history of two cesarean deliveries, who presented with reducible umbilical swelling and later experienced another swelling on the umbilicus, accompanied by cyclic umbilical pain and bleeding. The patient was diagnosed with an umbilical hernia and umbilical endometriosis, which was confirmed on histopathological evaluation.

Umbilical swelling can have several differential diagnoses, including benign and malignant tumors of the umbilicus. Benign diagnoses may include endometriosis, umbilical hernia, granuloma, hematoma, or lipoma. Malignant tumors may include primary malignant tumors of the umbilicus or metastatic tumors, such as Sister Joseph Mary nodules. Proper patient history, physical examination, and histopathological examination may help differentiate these diagnoses [2,4].

Endometriosis occurs in about 6% to 10% of women of reproductive age. Although endometriosis commonly affects pelvic organs, it can affect any organ in the body. The umbilicus is one of the organs that is less commonly affected [1,4].

The pathogenesis of endometriosis is not fully clear, but there are some theories that explain how endometriosis develops. The commonly recognized theory is retrograde menstruation or Sampson’s theory, which explains that ectopic implantation occurs after retrograde flow of endometrial cells and the spread through peritoneal circulation [1,4,7]. The others are lymphatic or hematogenous spread, which explains that ectopic implantation occurs after endometrial cells flow through the blood and lymphatic system. Metaplasia of embryonic remnants, which explains endometriosis, occurs after metaplasia of embryonic cells, such as umbilical vessel remnants or urachus. The last one is surgical seeding, which explains that ectopic implantation occurs after cells spread following abdominal surgery [4,8,9].

Primary endometriosis occurs spontaneously, while secondary endometriosis is seen after surgical interventions that penetrate or open the uterine cavity. The pathogenesis of endometriosis in our patient was likely due to surgical seeding. The patient had undergone two cesarean deliveries; procedures such as cesarean section may inoculate endometrial tissue in the abdominal wall during manipulation or stitching. Surgeons may need to pay attention to proper technique and surgical care to reduce the risk of secondary endometriosis. The risk can also be reduced by saline cleaning during surgery [7,10].

Clinical presentations of umbilical endometriosis include umbilical swelling and/or umbilical bleeding with pain, which increases with menstruation. Umbilical endometriosis may coexist with pelvic endometriosis in up to 40% of cases; therefore, a thorough evaluation of these patients is crucial [7,11].

Umbilical hernia is defined as a ventral abdominal hernia that occurs within 3 cm of the area of the umbilicus. It is usually diagnosed clinically by patient history and physical examination. Radiological examination may be required to rule out other diagnoses. Umbilical endometriosis and umbilical hernia may share similar clinical features, but when they occur together, which is very rare, they might present difficulties in making an accurate diagnosis [4,5].

Diagnostic laparoscopy is still considered the gold-standard method for the diagnosis of endometriosis, but it carries surgical risks and is expensive. Ultrasonography and MRI can be used for radiological evaluation, but their use does not replace diagnostic surgery. As MRI was not available in our setting, this patient was assessed preoperatively by ultrasonography. Ultrasonography carries the benefit of availability and affordability. It can be considered a first‐line investigation in conjunction with patient history and abdominal pelvic examination among women with suspected endometriosis. MRI has been shown to have a higher diagnostic accuracy than ultrasonography, but it is not recommended for routine use in women with suspected endometriosis [6,7].

Management options for endometriosis include medical and surgical therapy. Medical management does not cure the patient, but it provides relief from symptoms. Medications such as progestogens, oral contraceptive pills, or danazol can be used. Surgical excision is curative and has a low recurrence rate of less than 5%. Surgical excision also allows for histopathological examination of the excised tissue. It is therefore considered the gold-standard treatment option. Medical therapy might also be used as adjunct therapy for severe cases of endometriosis. As endometriosis has a chance of recurrence, follow-up of the patient after surgery is crucial [7,8].

## Conclusions

Umbilical swelling has several differential diagnoses, which can be either primary or metastatic disease of the umbilicus. Concurrent umbilical endometriosis and umbilical hernia are rare, and their clinical features can easily be confused, causing difficulties in making an accurate diagnosis. Cyclic umbilical swelling, pain, and umbilical bleeding should raise the suspicion of umbilical endometriosis. This case report emphasizes the importance of thorough history taking, physical examination, and histopathological examination for all women presenting with ambiguous umbilical swelling.
